# Supplementary material for: Effectiveness of Infection Control Teams in Reducing Healthcare-Associated Infections: A Systematic Review and Meta-Analysis
Source: Int J Environ Res Public Health. 2022 Dec 19;19(24):17075. doi: 10.3390/ijerph192417075 (PMC9779570; doi:10.3390/ijerph192417075)
Supplement: Supplementary file 1 [file ijerph-19-17075-s001.zip › Table S2. Eligibility criteria.pdf]

**Eligibility criteria**

| | **Inclusion** | **Exclusion** |
|---|---|---|
| Population/setting | Patients in inpatient hospitals or outpatient healthcare settings.<br>Residents in long-term care setting.<br>Any kind of healthcare professionals such as doctors, nurses, epidemiologists, microbiologists and nursing care home staff. | Non-healthcare professionals such as cleaning staff.<br>Healthcare professionals in non-clinical setting such as class room or learning laboratory |
| Intervention | All infection prevention and control measures by ICT including ICLN system such as<br>_ Formulating and revising policies/guidelines<br>_ Performing surveillance of HCAIs<br>_ Training and educating healthcare professionals<br>_ Monitoring and auditing practices and standard of care<br>_ Liaising with other staff and departments<br>The intervention can be of any duration and frequency. | _ Outbreak management<br>_ Infection control measures for human immunodeficiency virus, tuberculosis and malaria |
| Comparison | Any intervention or usual care | |
| Outcome | Patient-based/clinical outcomes<br>1. Rate of HCAIs | Rate of antimicrobial prescription |

| | | |
|---|---|---|
| | 2. Death due to HCAIs<br>3. Length of hospital stay<br>Staff-based/behavioural outcomes<br>1. Compliance with infection control practices<br>Additional outcomes<br>1. Cost of healthcare system or patients | |
| Study design | Randomised control trial (individual or cluster) | 1. Intervention studies (experimental) without control groups<br>2. Intermittent time series<br>3. Observational studies (nonexperimental)<br>4. Cohort (retrospective and prospective)<br>5. Case control<br>6. Case series<br>7. Cross-sectional<br>8. Ecologic<br>9. Reviews<br>10. Expert opinion or consensus<br>11. Editorial letter<br>12. Guideline<br>13. Implementation<br>14. Commentary<br>15. Outbreak reports |
| Period | No limitation | |
| Language | English language only | |
