# Supplementary material for: Effectiveness of Infection Control Teams in Reducing Healthcare-Associated Infections: A Systematic Review and Meta-Analysis
Source: Int J Environ Res Public Health. 2022 Dec 19;19(24):17075. doi: 10.3390/ijerph192417075 (PMC9779570; doi:10.3390/ijerph192417075)
Supplement: Supplementary file 1 [file ijerph-19-17075-s001.zip › Table S3. Search strategy.pdf]

Search strategy

PubMed

#1	Search ("health personnel"[MeSH] OR health personnel[tw] OR (health* NEAR/3 personnel) OR "patient care team"[MeSH] OR patient care team*[tw] OR health care team*[tw] OR healthcare team*[tw] OR "physicians"[MeSH] OR "physician*"[tw] OR "doctor*"[tw] OR "nurses"[MeSH] OR "nurse*"[tw] OR link nurse*[tw] OR liaison nurse*[tw] OR "practitioner*"[tw] OR "specialist*"[tw] OR health consultant*[tw] OR healthcare professional*[tw] OR health care professional*[tw] OR "pharmacists"[MeSH] OR "pharmacist*"[tw] OR "microbiologist*"[tw] OR "champion*"[tw] OR "team*"[tw] OR manager*[tw] OR "preventionist*"[tw])
#2	Search ("infection control"[MeSH] OR infection control[tw] OR (infection* NEAR/3 control) OR (infection* AND control) OR infection prevention[tw] OR infection management[tw] OR ("infections"[MeSH] OR "infection*"[tw] AND ("control*"[tw] OR "prevention*"[tw] OR "management"[tw])))
#3	Search (infection control team*[tw] OR infection control specialist*[tw] OR "infection control practitioners"[MeSH] OR infection control practitioner*[tw] OR infection control doctor*[tw] OR infection control nurse*[tw] OR infection control link nurse*[tw] OR infection control champion*[tw] OR infection control preventionist*[tw])
#4	Search ((randomized controlled trial [pt] OR controlled clinical trial [pt] OR randomized [tiab] OR placebo [tiab] OR clinical trials as topic [mesh: noexp] OR randomly [tiab] OR trial [ti]) NOT (animals [mh] NOT humans [mh]))
#5	Search (((#1 AND #2) OR #3) AND #4)
#6	Search (((#1 AND #2) OR #3) AND #4) Filters: English

EMBASE

#1	(health personnel OR (health* N3 personnel) OR patient care team* OR health care team* OR healthcare team* OR physician* OR doctor* OR nurse* OR link nurse* OR liaison nurse* OR practitioner* OR specialist* OR health consultant* OR healthcare professional* OR health care professional* OR pharmacist* OR microbiologist* OR champion* OR team* OR manager* OR preventionist*):ti,ab,kw
#2	MeSH descriptor: [Health Personnel] explode all trees
#3	MeSH descriptor: [Physicians] explode all trees
#4	MeSH descriptor: [Nurses] explode all trees
#5	MeSH descriptor: [Pharmacists] explode all trees
#6	MeSH descriptor: [Patient Care Team] explode all trees
#7	#1 OR #2 OR #3 OR #4 OR #5 OR #6
#8	(infection control OR (infection* NEAR/3 control) OR (infection* AND control) OR infection prevention OR infection management):ti,ab,kw
#9	MeSH descriptor: [Infection Control] explode all trees
#10	#8 OR #9
#11	#7 AND #10
#12	(infection control team* OR infection control specialist* OR infection control practitioner* OR infection control doctor* OR infection control nurse* OR infection control link nurse* OR infection control champion* OR infection control preventionist*):ti,ab,kw
#13	MeSH descriptor: [Infection Control Practitioners] explode all trees
#14	#12 OR #13
#15	#11 OR #14

Cochrane

#1	(health personnel OR (health* N3 personnel) OR patient care team* OR health care team* OR healthcare team* OR physician* OR doctor* OR nurse* OR link nurse* OR liaison nurse* OR practitioner* OR specialist* OR health consultant* OR healthcare professional* OR health care professional* OR pharmacist* OR microbiologist* OR champion* OR team* OR manager* OR preventionist*):ti,ab,kw
#2	MeSH descriptor: [Health Personnel] explode all trees
#3	MeSH descriptor: [Physicians] explode all trees
#4	MeSH descriptor: [Nurses] explode all trees
#5	MeSH descriptor: [Pharmacists] explode all trees
#6	MeSH descriptor: [Patient Care Team] explode all trees
#7	#1 OR #2 OR #3 OR #4 OR #5 OR #6
#8	(infection control OR (infection* NEAR/3 control) OR (infection* AND control) OR infection prevention OR infection management):ti,ab,kw
#9	MeSH descriptor: [Infection Control] explode all trees
#10	#8 OR #9
#11	#7 AND #10
#12	(infection control team* OR infection control specialist* OR infection control practitioner* OR infection control doctor* OR infection control nurse* OR infection control link nurse* OR infection control champion* OR infection control preventionist*):ti,ab,kw
#13	MeSH descriptor: [Infection Control Practitioners] explode all trees
#14	#12 OR #13
#15	#11 OR #14

CINAHL

S1	(MH "HealthPersonnel+") OR (MH"Multidisciplinary CareTeam+") OR (MH"Physicians+") OR (MH"Nurse Practitioners+") OR (MM "Pharmacist)
S2	TI (health personnel OR (health* N3 personnel) OR patient care team* OR health care team* OR healthcare team* OR physician* OR doctor* OR nurse* OR linknurse* OR liaison nurse* OR practitioner* OR specialist* OR healthconsultant* OR healthcare professional* OR health careprofessional* OR pharmacist* OR microbiologist* OR champion* OR team* OR manager* OR preventionist*
S3	AB (health personnel OR (health* N3 personnel) OR patient care team* OR health care team* OR healthcare team* OR physician* OR doctor* OR nurse* OR linknurse* OR liaison nurse* OR practitioner* OR specialist* OR healthconsultant* OR healthcare professional* OR health careprofessional* OR pharmacist* OR microbiologist* OR champion* OR team* OR manager* OR preventionist*
S4	S1 OR S2 OR
S5	(MH "Infection Control)
S6	TI (infection control OR (infection* N3 control) OR (infection* AND control) OR infection prevention OR infection management
S7	AB (infection control OR (infection* N3 control) OR (infection* AND control) OR infectionprevention OR infection management
S8	S5 OR S6 OR
S9	S4 AND S8
S10	(MM "Infection Preventionists")
S11	TI (infection control team* OR infectioncontrol specialist* OR infection control practitioner* OR infectioncontrol doctor* OR infection control nurse*OR infection control link nurse* OR infectioncontrol champion* OR infection control preventionist
S12	AB (infection control team* OR infection control specialist* OR infection control

	practitioner* OR infection control doctor* OR infection control nurse*OR infection control link nurse* OR infection control champion* OR infection control preventionist
S13	S10 OR S11 OR S12
S14	S9 OR S13
S15	((MH "randomized controlled trials") OR (MH "double-blind studies") OR (MH"single-blind studies") OR (MH "random assignment") OR (MH" pretest-post test design") OR (MH "cluster sample") OR TI (randomised OR randomized) OR AB (random*) OR TI (trial) OR (MH "sample size") AND AB (assigned OR allocated OR control) OR (MH "placebos") OR PT (randomized controlled trial) OR AB (control W5 group) OR (MH"crossover design") OR (MH "comparative studies") OR AB (cluster W3 RCT)) NOT (((MH"Animals+") OR (MH" animal studies") OR TI (animal model*)) NOT (MH "human")
S16	S14 AND S15
